# The role of intimate partner violence perpetrators' resting state functional connectivity in treatment compliance and recidivism

**DOI:** 10.1038/s41598-024-52443-3

**Published:** 2024-01-30

**Authors:** Ángel Romero-Martínez, María Beser, Leonor Cerdá-Alberich, Fernando Aparici, Luis Martí-Bonmatí, Carolina Sarrate-Costa, Marisol Lila, Luis Moya-Albiol

**Affiliations:** 1 Department of Psychobiology, University of Valencia, Valencia, Spain; 2 Biomedical Imaging Research Group (GIBI230), La Fe Health Research Institute, Valencia, Spain; 3 Department of Social Psychology, University of Valencia, Valencia, Spain

**Keywords:** Human behaviour, Neuroscience, Cognitive neuroscience

## Abstract

To expand the scientific literature on how resting state functional connectivity (rsFC) magnetic resonance imaging (MRI) (or the measurement of the strength of the coactivation of two brain regions over a sustained period of time) can be used to explain treatment compliance and recidivism among intimate partner violence (IPV) perpetrators. Therefore, our first aim was to assess whether men convicted of IPV (n = 53) presented different rsFC patterns from a control group of non-violent (n = 47) men. We also analyzed if the rsFC of IPV perpetrators before staring the intervention program could explain treatment compliance and recidivism one year after the intervention ended. The rsFC was measured by applying a whole brain analysis during a resting period, which lasted 45 min. IPV perpetrators showed higher rsFC in the occipital brain areas compared to controls. Furthermore, there was a positive association between the occipital pole (OP) and temporal lobes (ITG) and a negative association between the occipital (e.g., occipital fusiform gyrus, visual network) and both the parietal lobe regions (e.g., supramarginal gyrus, parietal operculum cortex, lingual gyrus) and the putamen in IPV perpetrators. This pattern was the opposite in the control group. The positive association between many of these occipital regions and the parietal, frontal, and temporal regions explained treatment compliance. Conversely, treatment compliance was also explained by a reduced rsFC between the rostral prefrontal cortex and the frontal gyrus and both the occipital and temporal gyrus, and between the temporal and the occipital and cerebellum areas and the sensorimotor superior networks. Last, the enhanced rsFC between the occipital regions and both the cerebellum and temporal gyrus predicted recidivism. Our results highlight that there are specific rsFC patterns that can distinguish IPV perpetrators from controls. These rsFC patterns could be useful to explain treatment compliance and recidivism among IPV perpetrators.

## Introduction

The estimated prevalence of intimate partner violence (IPV) across 161 countries indicates that approximately 27% of women, aged 15–49, have experienced some form of physical and/or sexual IPV, mostly from their male partner, throughout their lives^1^. Many factors have been identified as risks for IPV perpetration, such as personality disorders, executive dysfunctions, emotional dysregulation, drug misuse, emotional processing dysfunctions, and childhood trauma, among others^2,3^. However, much of this research is based on self-reports and/or qualitative interviews. Therefore, we need to take one step further to deepen our understanding of the underlying factors of IPV perpetration and use a combination and variety of techniques to properly design intervention programs.

Neuroscience has provided us with insights into the connection between neurobiological factors, like neuroimaging techniques, and the possible psychological dimensions linked to violent behavior. More specifically, structural and functional neuroimaging techniques have allowed us to deepen our understanding of violence. Applying these techniques has enabled the identification of brain circuits and/or areas with abnormal functioning in violent individuals^4–7^. The significance of these techniques lies in their ability to overcome the limitations inherent in the self-reports used in psychology, such as social desirability and exaggeration of symptoms, among others^8^.

The use of neuroimaging techniques, especially functional magnetic resonance imaging (fMRI), has enabled us to see how certain brain regions activate in response to certain tasks (task-dependent activation). However, there are also certain brain areas which show an increased activation during resting periods but remain deactivated during these demanding tasks^9^. Delving deeper into the topic of brain circuit activation during tasks and intrinsic resting-state activity could provide valuable and complementary information to better understand a complex phenomenon like violence^10^. In this regard, resting state functional connectivity (rsFC) refers to the analysis of the intrinsic activity when the brain is at rest, without any external stimulation. It is the measurement of the strength of the coactivation of two brain regions over a sustained period of time^11^. The activation of the two analyzed brain regions during a period of time could be positively or negatively correlated. By analyzing the differences in rsFC patterns between violent and non-violent individuals, it might be possible to establish risk profiles for violence proneness. It might even be possible to go further and assess if there is an intrinsic resting-state activity relevant to understanding why certain individuals tend to react with violence. In fact, two recent systematic reviews concluded that this type of analysis could be used as an indicator or trait for proneness to violence^12,13^.

However, before studying the brain functional connectivity underlying violence, it is important to identify the brain regions that show different activation in violent individuals when they process specific types of information. In this regard, the reduced activation of the dorsolateral prefrontal cortex (dlPFC), orbital frontal cortex (OFC), and anterior cingulate cortex (ACC) during emotional tasks (e.g., images, sounds, fear conditioning, etc.) may cause individuals with high scores on antisocial personality traits to be more prone to aggressive behavior because of inappropriate emotional processing^14^. The combination of reduced prefrontal functioning and increased limbic activity during emotional processing could also lead to this type of behavior. In this scenario, the violence linked to this imbalance could be attributed to high emotional content and impulsivity^15^. This model suggests that prefrontal regions (control system) are unable to regulate limbic irritability, which facilitates behavioral instability and violence under uncertain circumstances^12,13,15^. However, it is important to understand whether this imbalance is also applicable during resting periods or only during emotional processing processes. Moreover, it is also important to consider other brain circuits during resting periods when characterizing violent individuals. That is, to go beyond the connections between the prefrontal regions and the amygdala or other limbic structures, and also consider other circuits that could be potentially relevant to violent tendencies due to their role in facilitating violence (e.g., emotion regulation, processing, among others).

When it comes to characterizing violent populations, a reduced rsFC between the left dlPFC and the basal basolateral amygdala (bilaterally) and between the OFC and the cerebellar vermis may be a characteristic of violent individuals. This diminished rsFC connectivity could be interpreted as vigilance towards potentially threatening visual cues^16,17^. Furthermore, a heightened rsFC between the amygdala and both the inferior frontal gyrus and the left superior temporal gyrus has been shown to entail high anger traits in violent individuals characterized by impulsive aggression^17^. In addition, the amygdala and the ACC have been found to maintain a high rsFC with the right cerebellar hemisphere, the fusiform and lingual gyrus (LG), the cuneus and precuneus calcarine cortex, and the superior occipital cortex in impulsive and violent offenders^16^. Furthermore, an increased rsFC between the supramarginal gyrus (SMG) and adjacent areas has been shown to distinguish juvenile offenders from non-violent individuals. The authors suggest that such connectivity could be considered a sign of brain immaturity in juvenile offenders^18^. There has also been evidence regarding alterations in the default mode network (DMN) in violent juvenile offenders when compared to normative young adults. More specifically, compared to controls, the violent group exhibited a decreased rsFC in the right middle temporal gyrus, left angular gyrus (AG), right precuneus, and right middle frontal cortex, but an increased connectivity in the posterior cingulate cortex (PCC) and the above-mentioned brain regions^19^. The authors suggested that these patterns of rsFC might be responsible for difficulties in maintaining self-regulation.

In terms of IPV perpetrators, only two studies have assessed whether the rsFC of IPV perpetrators differed from non-violent individuals. The first one concluded that IPV perpetrators exhibited an increased and negative rsFC between the left posterior cerebellar Crus II and the left parahippocampus/hippocampus and an increased and positive rsFC between the right posterior cerebellar Crus II and the right precuneus, right AG, left PCC, and bilateral parahippocampal when compared to controls. They also exhibited an increased and negative rsFC between the right lateral cerebellar Crus II and the mid-temporal gyrus compared to controls^20^. The other study reported that, compared to controls, IPV perpetrators exhibited a higher rsFC between the right basolateral amygdala and the temporal pole; left ventrolateral prefrontal cortex (vlPFC) and brainstem, middle temporal area, hippocampus; left dlPFC and putamen-caudate; right posterior insula and putamen; left posterior insula and the AG and middle temporal area. However, IPV perpetrators also exhibited a lower rsFC between the right centromedial amygdala and the intraparietal, fusiform gyrus (FG), and occipital area; right ventrolateral PFC and sensorimotor area, premotor area, intraparietal sulcus, and occipital area when compared to controls^21^. Due to the wide variety of studies that identify different patterns of brain connectivity in violent individuals, including IPV perpetrators, it would be appropriate to conduct a whole brain analysis instead of focusing only on specific brain areas. This broader perspective would help clarify whether there are reliable rsFC patterns across samples that affect not only prefrontal and limbic areas, but also other less explored areas.

Although these differentiating rsFC patterns offer valuable information to clarify and adequately understand a complex phenomenon such as IPV, they do not provide too much information on how to prevent dropout (or a premature abandonment of psychotherapeutic treatment designed for men convicted of IPV perpetration to prevent future IPV) and recidivism (or reoffending). One of the two main problems that arise when dealing with IPV perpetrators is that many IPV perpetrators do not complete intervention programs designed to reduce recidivism (or reoffending), which considerably increases the risk of repeated IPV^22,23^. Only one study has measured brain activation during the resting period, rather than focusing on rsFC, in combination with other factors such as drug misuse, personality traits, and demographic factors, among others, to explain recidivism. The conclusions from this study indicated that a diminished activation of the parietal lobe (bilaterally) and right cerebellar, as well as a heightened activation of the temporal lobe (bilaterally), were considerable predictors of recidivism^24^. However, if we take a look at other areas of research that contemplate the usefulness of rsFC, it can be stated that rsFC could help explain treatment compliance. This is based on a previous study which concluded that the measurement of rsFC of certain brain areas helped explain the psychotherapy response of patients diagnosed with posttraumatic stress disorder, by distinguishing those who responded to therapy from those who did not^25^.

### Current study

To our knowledge, only a limited number of studies have measured whether individuals who commit IPV, as violent individuals, have different rsFC compared to non-violent individuals. Furthermore, no studies have explored whether the rsFC of IPV perpetrators could explain treatment compliance and recidivism. Therefore, the main objective of this study was twofold. First, we tried to replicate the differences in specific rsFC between IPV perpetrators and non-violent individuals (control group). Based on previous empirical research on rsFC patterns in non-violent and violent individuals, including IPV perpetrators^16–21^, we hypothesized that IPV perpetrators would present a negative (or inverse correlation coefficient) rsFC between the PFC and the limbic structures and cerebellum, as well as a positive correlation between the limbic structures (amygdala and hippocampus) and the temporal and occipital pole and cerebellum. Second, we aimed to assess whether the rsFC of IPV perpetrators would explain treatment compliance (dropout) and recidivism. Thus, we formulated a hypothesis based on previous conclusions in this field which have suggested that a reduced activation of the parietal and cerebellum and a heightened activation of the temporal lobes might explain the highest risk of recidivism^24^ and that a reduced rsFC between the PFC and the limbic areas might explain the highest emotional instability and violence proneness^12,13^. We, therefore, expected that the reduced rsFC between these brain areas would signal the highest dropout and IPV recidivism rates and/or risk.

## Methods

### Participants

The calculation of the necessary sample size to conduct this study established a minimum of 87 participants. This estimation included a confidence level of 95%, a margin of error of 5%, and the assumption of a population proportion of 6%^26^. From an initial sample of 105 healthy men that showed interest in our study, only 100 men were finally included after the screening process (4 of them did not complete the study, and one was eliminated from the statistical analyses because his IQ was below 80).

The IPV perpetrator group was recruited from the psychological and psychoeducational community treatment program “CONTEXTO”, which operates in the Department of Social Psychology at our University. This program is mandatory for men who receive a sentence of less than two years in prison for gender violence in their intimate partner relationships and who have no previous criminal records. The judicial system tends to offer this alternative on the condition that they complete the intervention program^27^. In addition, to participate in this study, individuals had to be free from any physical issues (e.g., brain damage, chronic pain, mild and/or severe cranioencephalic trauma with a temporary loss of consciousness lasting from minutes to days) or mental disorders (mood, personality, psychotic disorders, etc.), and have an IQ equal to or above 80. All participants were interviewed by two mental health professionals with considerable expertise regarding IPV perpetrators to verify these conditions. To be included in this study, the inter-rater reliability (Cohen's kappa) had to be above 0.70 in each of the conditions mentioned above. Moreover, to avoid artifacts or problems when acquiring the rsFC with an fMRI, participants needed to not have class III obesity (body mass index > 40), cranioencephalic metallic implants, and/or agoraphobia. The experimental group was finally composed of 53 heterosexual men convicted of IPV.

The control group was composed of 47 men who had no previous criminal records (including IPV or any kind of violence). The recruitment of the control group was based on advertisements published in the city of Valencia (Spain), and several social media posts. As a result, men who showed interest in participating in our study were first contacted via e-mail. Subsequently, an initial interview was arranged for screening purposes. The inclusion criteria for this control group were not having a criminal record of violence against their partner or another individual, which was verified based on a criminal record certificate issued by a public institution; and scoring below one on the Conflict Tactics Scale-II^28,29^.

Finally, the experiment was carried out following the ethical and legal guidelines of the Helsinki Declaration and was approved by the University of Valencia Ethics Committee (code: H1515749368278).

### Procedure

To conduct the study, each participant was initially screened. Afterwards, they had to attend one session at the psychobiology laboratories of the Faculty of Psychology (University of Valencia, Spain) and then a second session at the La Fe Health Research Institute (Valencia, Spain).

The men interested in being part of the study were interviewed via telephone to assess suitability before being allowed to participate. Afterwards, those who were not excluded and agreed to participate, signed an informed consent form and were also given an appointment for the session at the psychology laboratories.

During the first session and after signing the informed consent, a semi-structured individual interview was conducted with all participants to exclude those who did not meet the inclusion criteria and to collect the necessary sociodemographic data, psychological variables, and drug consumption information. This took place between 10 a.m. and 2 p.m. to minimize possible effects of fatigue later in the day.

Finally, once the previous session had finished, the participants were called back within one week to perform an fMRI at the University and Polytechnic Hospital of La Fe. During the session, the participants were introduced to the fMRI and were asked to "not think about anything"^30^. This session lasted approximately 45 min and no additional tasks were programmed for immediately before or after this measurement. At the end of this phase, one day after the fMRI acquisition, the individual was thanked for their participation and received €100 to cover dietary and travel expenses.

All these phases described above took place during the evaluation phase of the intervention program. Regarding dropout and recidivism, dropout was monitored constantly during the ongoing treatment, and risk of recidivism was calculated after the intervention ended, approximately nine months after the initial interview and the fMRI. Additionally, official recidivism was obtained for the year following the end of the IPV perpetrators' treatment.

### Instruments

The calculation of the intelligence quotient (IQ) was based on the application of the Spanish validated version of the Kaufman Brief Intelligence Test^31^. It has been previously noted that this instrument is effective at measuring both verbal and nonverbal intelligence with good sensitivity and specificity^32^ and has been applied to measure these in IPV perpetrators and non-violent men^33,34^.

#### Alcohol and drug misuse

For this study, we employed the Alcohol Use Disorders Identification Test (AUDIT)^35^ conveniently adapted and validated to Spanish^36^ to assess the presence of alcohol misuse. The higher the score, the higher the risk of having an alcohol misuse or use disorder. We also interviewed participants to calculate the number of units of alcohol (1 g = 1 UA) they used per day. This instrument is effective at screening individuals and differentiating those who exhibit a pathological alcohol use or alcohol misuse from those who do not^37^. Furthermore, it has been widely employed for measuring alcohol use patterns in IPV perpetrators^38,39^.

To assess whether participants exhibited cannabis and/or cocaine misuse we employed the Severity Dependence Scale (SDS)^40^ adapted to Spanish for each drug^41^. This test consists of five items, which range from 0 (never/almost never) to 3 (always/nearly always), with a cut-off score of 5. Furthermore, the number of joints per day as well as the amount of cocaine (number of grams of cocaine per week) used by the participants was registered. This tool shows a high level of sensitivity and specificity to distinguish between individuals who have misused cannabis and cocaine and those who have not^42,43^. Furthermore, it has been previously employed with IPV perpetrators to measure cannabis and cocaine misuse^33,34^.

To measure the levels of IPV, a widely employed tool in this field of research was used; the Revised Conflict Tactics Scale (CTS2)^29^ adapted to Spanish^28^. The adapted Spanish version has been proven to be suitable at differentiating IPV perpetrators and non-IPV perpetrators. It has also been significantly related to variables that increase the risk of this kind of violence (e.g., impulsivity, drug misuse, among others)^27,38,39^.

#### Treatment compliance

To assess this variable, we considered two variables of a different nature: dropout as a dichotomized variable and intervention dose as a quantitative variable. Participants were categorized as *completers* (0) when they finished the intervention, or *dropout* (1) if they abandoned the intervention before it ended. We completed the data by including *intervention dose* (i.e., number of intervention sessions participants attended). We calculated the percentage of intervention dose that participants completed, ranging from 0 to 100.

To measure recidivism, we also included a dichotomized and a quantitative variable. The rate of *official IPV recidivism* was obtained during the year after the IPV perpetrators' treatment ended. This data was provided by the monitoring system of the Spanish Ministry of the Interior (responsible for the penitentiary system), concretely from the VioGén database. Participants who did not reoffend were classified as 0 and those who reoffended were assigned a 1. To calculate the risk of recidivism, we employed the validated Spanish version^44^ of the *Spousal Assault Risk Assessment Guide* (SARA)^45^. This questionnaire contains 20 items, which range from 0 (= *absence*) to 2 (= *presence*). This test measures several questions related to IPV. A high score is interpreted as high risk of recidivism. In the same way as with treatment compliance, we calculated the percentage of risk of recidivism (ranging from 0 to 100 the risk of recidivism). It has been previously established that the SARA presents good predictive validity^46^.

### fMRI data acquisition and analysis

The fMRI data were acquired on a 3 T magnet (Achieva TX, Philips Healthcare Best, The Netherlands) using an 8-channel head coil with parallel acquisition technology (SENSE). Participants were informed beforehand of the need to avoid moving while conducting the fMRI. The acquisition protocol consisted of a T1-weighted high spatial resolution 3D gradient echo sequence with the following parameters: echo time = 3 s, repetition time = 6.2 s, twist angle = 100, voxel size = 1 × 1 × 1 mm^3^, and 6 min long. The T2*-weighted 2D EPI BOLD (blood oxygen level dependent) rsfMRI sequence was acquired with the following main parameters TE = 35 ms, TR = 2000 ms, temporal dynamics = 265, pixel size = 1.8 × 1.8 mm^2^, slice thickness = 5 mm, and total duration of 9 min.

The functional images (rs-fMRI) were realigned, slice-time corrected, normalized to MNI space, and smoothed. More specifically, the rs-fMR images were realigned by registering each one to the first slice of the first session, using b-spline interpolation. The temporal mismatch was corrected by shifting the functional images in time and resampling them by sinc interpolation. The ART-repair software was used as a method of detecting artifacts, considering acquisitions that showed a mean image shift greater than 0.9 mm or global changes in the BOLD signal greater than 5 standard deviations as possible outliers.

The functional and anatomical images (T1) were normalized and segmented separately, using the mean BOLD signal (z-score) as the reference image for the functional image, and the T1 image for the anatomical image. Normalization was carried out by registering each image spatially on a standardized MNI space with isotropic voxels of 2 mm for functional data and 1 mm for anatomical data.

The region of interest (ROI)-by-ROI connectivity analysis allowed us to obtain the level of functional connectivity between each pair of brain areas.

The brain regions used correspond to 91 cortical and 15 subcortical regions defined by the Harvard–Oxford FSL probabilistic atlas, and 26 cerebellar regions defined by the Automated Anatomical Labeling (AAL) atlas.

### Data analysis

After exploring whether demographic data, IQ, and drug misuse were normally distributed with the Kolmogorov–Smirnov test, *t*-test and Chi-square analyses were conducted to assess any group differences. Those that differed across groups were included as covariates in the regression analysis.

Regarding the first objective of this study, individual correlation matrices were entered into a second-level general linear model. We compared the rsFC of the IPV perpetrator and control scans using a logistic regression analysis. The correlation maps between significant regions were adjusted using two thresholds, one at the connection level (intensity of the relationship between ROIs) and another at the cluster or grouping level^47,48^, with a connection threshold of p < 0.05 corrected by false discovery rate (FDR). From these correlation maps, the differences in connectivity between groups (IPV perpetrators and controls) were evaluated for all pairs of brain regions using the general linear model (GLM), controlling the effect of those demographic and/or drug misuse variables that differed from groups by including them as covariates.

To calculate the effect of group and other interest variables on connectivity, a logistic regression with Lasso regularization was conducted, using train-test cross-validation, from the scikit-learn Python library^49^ and following the methodology found in previous studies^50,51^. This is a supervised learning algorithm used for binary classification problems.

With regard to the second aim of this research, the individual correlation matrices between significant regions were adjusted using a threshold at the connection level (intensity of the relationship between ROIs) p < 0.05 corrected by FDR. From these correlation maps, the effect of dropout and recidivism on IPV perpetrators' connectivity was evaluated using a logistic regression analysis. As stated in certain meta-analyses^52,53^, the effect of alcohol, and cocaine misuse were controlled when conducting regression models to predict dropout and recidivism.

For regression models, precision and accuracy metrics were used to explain the variance of the model. Precision refers to the model's ability to correctly predict the classes of observations. In the case of a binary logistic regression model, it calculates the proportion of true positive and true negative predictions relative to the total number of observations. Furthermore, accuracy is the ratio of correctly predicted binary outcomes (true positives and true negatives) to the total number of cases. It is expressed as a percentage, where higher accuracy indicates better performance.

All statistical analyses were conducted using the CONN 20b toolbox^54^ second-level analysis, from MATLAB (MathWorks, Inc., Natick, MA, United States).

### Ethics declarations

The experiment was carried out following the ethical and legal guidelines of the Helsinki Declaration and was approved by the University Ethics Committee (code: H1515749368278).

## Results

We initially checked whether there were any differences in demographic variables, IQ, and alcohol and drug misuse between groups. Participants did not differ in age, nationality, level of education or laterality, but there were differences found in alcohol and cocaine misuse. Specifically, a higher percentage of IPV perpetrators exhibited higher alcohol and cocaine misuse compared to the control group. Even though they did not differ in the amount of drug consumption (Table 1) we still considered it appropriate to include both variables as covariates in ulterior analysis when calculating group differences.

**Table 1:** Means, standard deviations, percentages, and means comparisons for socio-demographic and psychological variables.

	IPV perpetrators (*n* = 53)	Controls (n = 47)	*t*-test/Chi-square	Significance
Age (*M, SD*)	41.13 (11.73)	36.87 (12.24)	1.78	0.079
Nationality (%)				
Spanish	92	89	3.86	0.145
Latin Americans	0	5		
Others	8	6		
Level of education (%)				
Primary/lower secondary	57	53	3.14	0.208
Upper secondary/vocational training	36	43		
University	7	4		
Laterality				
Right-handed	91	87	2.38	0.498
Left-handed	9	13		
IQ	97.24 (7.93)	100.31 (12.08)	− 1.49	0.139
Alcohol misuse (%)				
Yes	34	6	11.42	0.001
No	66	94		
Units of alcohol per day	0.48 (0.69)	0.30 (0.46)	1.61	0.111
Cannabis misuse (%)				
Yes	15	4	3.25	0.071
No	85	96		
Number of joints per day	1.59 (2.17)	1.31 (2.21)	0.370	0.714
Cocaine misuse (%)				
Yes	9	-	4.67	0.031
No	91	100		
Grams per week	0.38 (0.90)	0.14 (0.22)	1.26	0.217
Dropout (%)				
Yes	27	–		
No	73			
Intervention dose	0.648 (0.307)	–		
Total risk of recidivism score	0.204 (0.094)	–		
Official recidivism (%)				
Yes	10	–		
No	90			

*IPV* intimate personal violence, *M* mean, *IQ* intelligent quotient, *SD* standard deviation.

### Group differences

**Figure 1:**
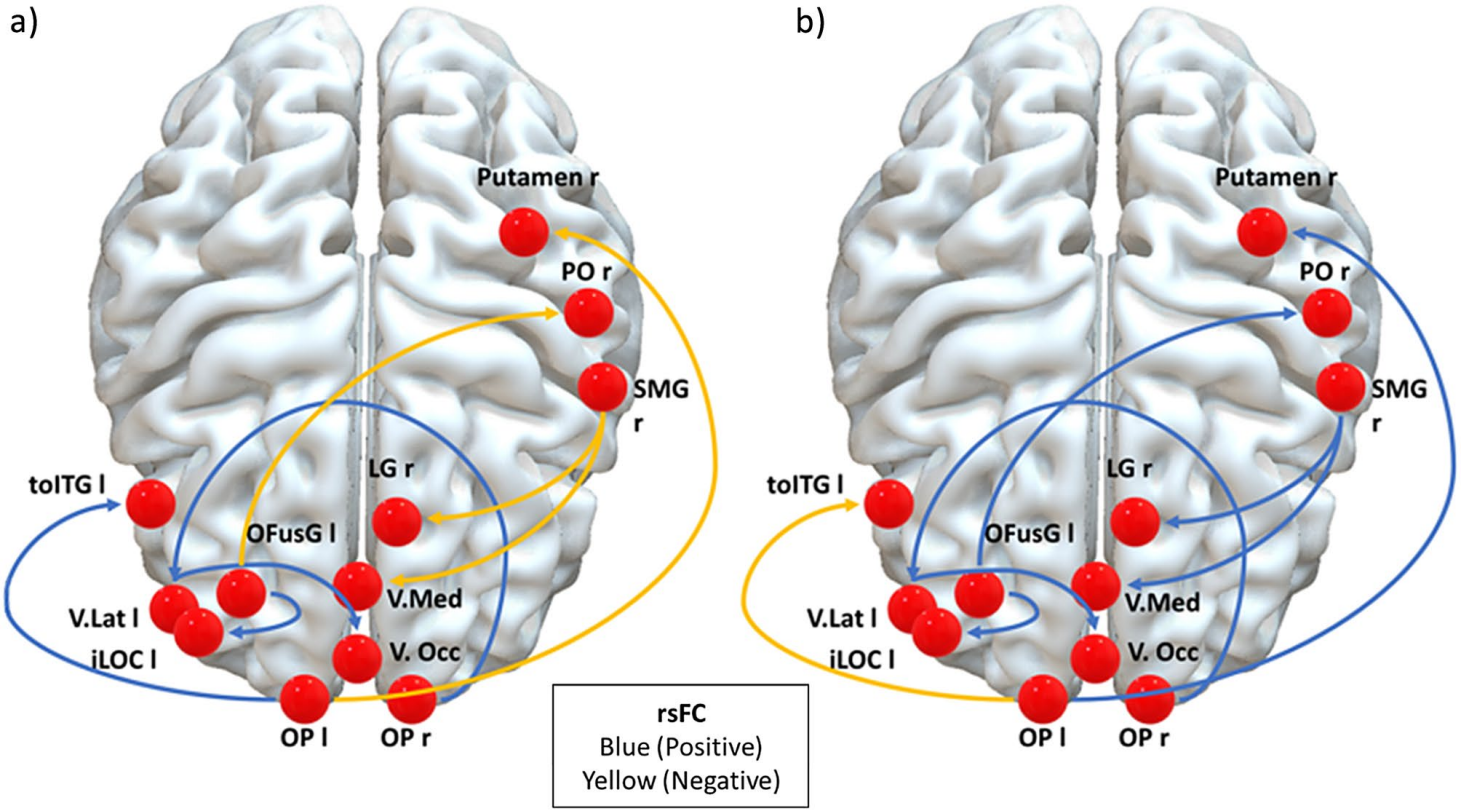
Significant differences in resting state functional connectivity (positive in blue colour and negative in yellow colour) patterns in intimate partner violence perpetrators (**a**) regarding controls (**b**). *iLOC* inferior division lateral occipital cortex, *LG* lingual gyrus, *OFusG* occipital fusiform gyrus, *OP* occipital pole, *PO* parietal operculum cortex, *rsFC* resting state functional connectivity, *SMG* supramarginal gyrus, *toITG* inferior temporal gyrus, temporooccipital part.

Regarding the first aim of this study, the percentage of explained variance was 0.60, with a precision of 0.89 for IPV perpetrators and 0.91 for controls. The analysis of rsFC revealed that IPV perpetrators showed a higher and positive rsFC between the occipital brain areas compared to controls. Additionally, IPV perpetrators exhibited a positive association between the occipital (OP) and temporal lobes (toITG) and a negative association between the occipital and the parietal lobe areas and putamen. Controls presented opposite patterns (Table 2 and Fig. 1). The cumulative power, representing the overall statistical power across all analyses, for the first objective was 0.95.

**Table 2:** Resting-state functional connectivity of IPV perpetrators and controls classified according to IPV perpetrators patterns.

Region1	Region2	IPV perpetrators	Controls	Beta contrast	p-unc	p-FDR	IC 95%	Statistic
Enhanced rsFC								
OFusG left	iLOC left	0.77	0.55	0.22	0.0005	0.0404	[− 0.29, − 0.16]	− 3.61
OP right	Visual lateral left (− 37, − 79, 10)	0.55	0.26	0.29	0.0003	0.0459	[− 0.36, − 0.22]	− 3.78
OP left	toITG left	0.12	− 0.09	0.21	0.0004	0.0312	[− 0.26, − 0.15]	− 3.69
Visual lateral left (− 37, − 79, 10)	Visual occipital (0, − 93, − 4)	0.62	0.37	0.26	0.0001	0.0213	[− 0.32, − 0.20]	− 3.99
Diminished rsFC								
OFusG left	PO right	− 0.09	0.11	− 0.21	0.0001	0.0243	[0.16, 0.26]	3.96
OP left	Putamen r	− 0.17	0.02	− 0.19	0.0001	0.0229	[0.14, 0.24]	3.97
Salience SMG right (62, − 35, 32)	LG right	− 0.20	0.05	− 0.25	0.0002	0.0233	[0.19, 0.32]	3.93
Salience SMG right (62, − 35, 32)	Visual medial (2, − 79, 12)	− 0.19	0.01	− 0.21	0.0003	0.0233	[0.16, 0.27]	3.77

*iLOC* inferior division lateral occipital cortex, *LG* lingual gyrus, *OFusG* occipital fusiform gyrus, *OP* occipital pole, *PO* parietal operculum cortex, *rsFC* resting state functional connectivity, *SMG* supramarginal gyrus, *toITG* inferior temporal gyrus, temporooccipital part.

### Treatment compliance

With regard to the second objective of this study, several significant rsFC connections emerged to explain dropout. A 0.25 of explained variance was obtained, with an accuracy of 0.85 and a precision of 0.80, for those who did not drop out and 1.00 for those who dropped out. Dropout was mainly explained by the positive association among occipital regions as well as the positive association between occipital regions and parietal, limbic and frontal regions. Furthermore, it was also explained by the inverse association between frontal (e.g., paracingulate gyrus; PaCiG) and temporal regions (posterior superior temporal gyrus; pSTG) (Table 3).

**Table 3:** Resting state functional connectivity as predictors of dropout, intervention dose, official recidivism, and total risk of recidivism of IPV perpetrators controlling the effect of alcohol and drug misuse.

Region1	Region2	Beta	p-unc	p-FDR	IC 95%	Statistic
Dropout						
pSMG right (parietal)	OFusG left (occipital)	0.25	0.0001	0.0145	[0.19, 0.30]	4.28
AG right (occipital)	LG left (occipital)	0.27	0.0006	0.0481	[0.20, 0.34]	3.69
AG right (occipital)	Networks visual lateral left (− 37, − 79, 10)	0.28	0.0004	0.0481	[0.21, 0.35]	3.79
LG left (occipital)	Precuneous cortex (parietal)	0.27	0.0009	0.0359	[0.19, 0.34]	3.55
LG left (occipital)	DMN LP right (47, − 67, 29) (parietal)	0.27	0.0001	0.0203	[0.21, 0.34]	4.18
LG left (occipital)	DMN PCC (1, − 61, 38) (limbic)	0.28	0.0005	0.0321	[0.22, 0.36]	3.71
DMN LP right (47, − 67, 29) (parietal)	LG left (occipital)	0.27	0.0001	0.0203	[0.21, 0.34]	4.18
Networks visual lateral left (− 37, − 79, 10) (occipital)	MidFG right (frontal)	0.31	0.0006	0.0492	[0.23, 0.39]	3.67
PaCiG right (frontal)	pSTG right (59, − 42, 13) (temporal)	− 0.27	0.0001	0.0193	[− 0.34, − 0.21]	− 4.19
Intervention dose (percentage)						
MidFG right (frontal)	pMTG right (temporal)	0.44	0.000225	0.0374	[0.362, 0.521]	3.99
pMTG right (temporal)	AG right (parietal)	0.50	0.00107	0.0444	[0.402, 0.601]	3.48
Networks salience RPFC left (− 32, 45, 27) (PFC)	Networks visual lateral left (− 37, − 79, 10) (occipital)	− 0.47	0.000282	0.0468	[− 552, − 0.383]	− 3.92
Networks salience RPFC right (32, 46, 27) (PFC)	aMTG left (temporal)	− 0.43	0.000004	0.0007	[− 0.50, − 0.36]	− 5.21
MidFG right (frontal)	pITG left (temporal)	− 0.36	0.000581	0.0483	[− 0.43, − 0.29]	− 3.68
pMTG right (temporal)	OFusG right (occipital)	− 0.30	0.000415	0.0344	[− 0.36, − 0.24]	− 3.79
pMTG right (temporal)	Networks visual occipital (0, − 93, − 4) (occipital)	− 0.35	0.001057	0.0444	[− 0.43, − 0.28]	− 3.49
Cerebelum Crus1 right	Cerebelum Crus2 right	− 0.59	0.000217	0.036	[− 0.70, − 0.49]	− 4.00
Cerebelum 3 right	Networks SensoriMotor Superior (0, − 31, 67)	− 0.27	0.000241	0.0401	[− 0.32, − 0.23]	− 3.97
Official recidivism						
aITG right	TOFusC left	0.43	0.0002	0.0302	[0.36, 0.49]	4.05
Total risk of recidivism (percentage)						
OFusG right	Cerebelum Crus1 left	0.80	0.000029	0.0049	[0.74, 0.86]	4.61
OFusG right	Vermis 9	0.72	0.000437	0.0362	[0.66, 0.79]	3.78

*AG* angular gyrus, *DMN* default mode networks, *pITG* inferior temporal gyrus, posterior división, *aITG* inferior temporal gyrus, anterior división, *LG* lingual gyrus, *LP* lateral parietal, *MidFG* middle frontal gyrus, *aMTG* middle temporal gyrus, anterior división, *pMTG* middle temporal gyrus, posterior división, *OFusG* occipital fusiform gyrus, *PaCiG* paracingulate gyrus, *PCC* posterior cingulate cortex, *RPFC* rostral prefrontal cortex, *pSMG* supramarginal gyrus, posterior división, *pSTG* posterior superior temporal gyrus, *TOFusC* temporal occipital fusiform cortex.

Not surprisingly, for the percentage of treatment compliance, the regression analysis revealed that the model's accuracy was 0.68, with a 0.26 of explained variance. In fact, treatment compliance was mainly explained by the negative association between occipital regions and temporal areas, frontal and temporal, and between cerebellum areas and sensorimotor superior networks (Table 3).

Regarding power calculation, a power of 0.58, and 0.87 were found for the dropout and percentage of treatment compliance, respectively.

### Recidivism

Regarding official recidivism (obtained during the year after the IPV perpetrators' treatment ended), the accuracy of this model was 0.75, with a precision for detecting non-recidivist of 0.82 and 0 for recidivist. In fact, recidivism was explained by the positive association between the inferior temporal gyrus, anterior division (aITG) and the temporal occipital fusiform cortex (TOFusC) (Table 3).

Regarding total risk of recidivism, the accuracy of rsFC in predicting this variable (calculated nine months after the fMRI acquisition) was 0.0056. In this sense, the positive association between the occipital and both the cerebellum and temporal gyrus predicted recidivism (Table 3).

The power calculation for recidivism was found to be 0.70 and 0.99 for official recidivism and total risk of recidivism, respectively.

## Discussion

Our results indicate that IPV perpetrators exhibit higher rsFC between the occipital brain areas (Ofus, iLOC, OP, and networks visual lateral) compared to controls. Inverse patterns between both groups were also observed. Concretely, IPV perpetrators exhibited a positive association between the occipital (OP) and temporal lobes (toITG) and a negative association between the occipital (e.g., occipital fusiform gyrus [OfusG left], visual network) and parietal lobe regions (e.g., SMG, PO, LG) and putamen, with controls showing opposite patterns. In terms of treatment compliance and recidivism, it is important to note that the percentage of explained variance associated with rsFC was higher for treatment compliance than for recidivism. Specifically, there was an increase of rsFC between the occipital (OfusG, LG, and visual lateral networks) and parietal brain regions (pSMG, AG) and between the occipital (visual lateral networks) and frontal areas (MidFG). In addition, there was a reduced rsFC between the frontal part of the corpus callosum (PaCiG) and the temporal lobe (pSTG right).

### Group differences

Regarding the first aim of this study, our results show that IPV perpetrators exhibit a differentiated rsFC pattern compared to controls. Concretely, IPV perpetrators show increased rsFC among occipital brain regions (OfusG, iLOC, OP, visual lateral, and visual occipital) and between the occipital (OP) and temporal areas (toITG), but a diminished rsFC between the occipital regions (LG and visual medial) and the parietal brain (Salience SMG) areas and putamen. Contrary to our expectations, these patterns did not correspond with those previously stated by empirical studies with violent men and IPV perpetrators^16–21^. Differences across studies could be attributed to methodological questions, for example, the reduced sample sizes employed in previous studies, or the type of tools used to measure rsFC.

Specific brain areas, such as the toITG, OfusG, and LG^18,21^, have enabled scientists to distinguish between IPV perpetrators or violent juvenile offenders and control subjects. These brain regions tend to be related to the integration of language and visual information, their recognition, and verbal fluency^55,56^. Curiously, a previous study that examined how IPV perpetrators react to images with different emotional content, revealed that IPV perpetrators showed an increased activity of several brain regions, such as the ITG, OfusG and LG, when processing threatening emotional images and images of women victims of IPV, compared to the control group^57^. All these results suggest that brain models assessing the rsFC profile of violent individuals should not only focus on the association between PFC and the limbic structures (i.e., amygdala and hippocampus). As highlighted by this study, research should go further and consider other brain regions and their connections, such as those associated with occipital and temporal brain areas. For example, some of those brain regions might be part of the fear conditioning pathway. Sensory cortices (e.g., temporal, and occipital areas) maintain connections with the amygdala (lateral nucleus), which distribute these signals to other nuclei of the amygdala. The signals are then projected to the brainstem, which regulates autonomic control (heart rate, etc.)^58^. These brain regions could contribute to IPV perpetration by being involved in processes such as decoding or regulating emotions, which in turn, might affect violence proneness.

Our data indicated that certain connectivity patterns of IPV perpetrators were opposite to those of the control group. In this regard, IPV perpetrators exhibited higher connectivity among the occipital cortex areas and a reduced rsFC between the occipital and the temporal and parietal areas. Attending to these results, and in combination with the conclusions pointed out by Lee et al.^57^ and the importance of the ITG and OfusG for facial processing^59–61^, we can speculate that these regions, which underlie the visual and verbal encoding of social relevant information such as faces, constitute one of the foundations for explaining dysfunctional schemas and cognitive distortions present in some IPV perpetrators. These theories establish that IPV proneness might be explained, at least in part, by the maintenance of these cognitive distortions (e.g., sexism, hostile attributions to other intentions, and self-schemas, among others)^62^ and a failure to recognize faces expressing emotions^33^. Therefore, it would be relevant to measure whether this intrinsic resting-state activity is linked to previous research in this field. Concretely, if it is connected to alterations in emotion decoding processes or other cognitive alterations such as executive dysfunctions, which have been related to IPV proneness^33,34^. Furthermore, we emphasize the importance of analyzing this spontaneous brain activity along with other relevant factors of IPV perpetration, such as emotion regulation, which has also been linked to IPV perpetration^3^. Future research should explore whether these brain regions allow distinguishing IPV perpetrators from non-violent men and whether they precede violence intake facilitated by a previous hostile interpretation of the surrounding environment.

### Treatment compliance

Regarding the second aim of this study, we investigated whether rsFC could explain treatment compliance and recidivism, using both dichotomized and quantitative variables. It is important to keep in mind that the accuracy and precision of the statistical models were slightly better for treatment compliance than recidivism. Therefore, we could conclude that rsFC was a better predictor of treatment compliance than recidivism, regardless of the type of measurement (dichotomized or continuous). Once again, the occipital brain areas were excessively highlighted as important predictors of these variables, with positive rsFC (e.g., LG, AG, OfusG and networks visual lateral), contrary to our expectations. As stated above, much of these areas are related to visual and language processing (e.g., faces) and their integration^55,56,59^. Therefore, we could hypothesize that the positive association between the activation of these regions (AG, LG, and visual lateral) and between these areas and the parietal (precuneus cortex and LP), frontal (MidFG) and temporal (MTG) regions might explain the reduced ability to process new information and deal with it effectively.

This increased rsFC should be taken into account with other decrease patterns to explain treatment compliance. Concretely, there was a reduced rsFC between the rostral PFC (RPFC) and the frontal cortex (PaCiG) and both the occipital (visual lateral) and the temporal areas (STG, MTG and ITG), as well as between the temporal (MTG) and the occipital (OfusG), cerebellum areas and sensorimotor superior networks. Special attention should be directed to the RPFC and its negative associations with the occipital and temporal brain regions. In this regard, we would like to highlight the role of the RPFC when it comes to attention, self-generated representations (inner thoughts), prospective memory, and inhibitory control^63,64^. Furthermore, the precuneus cortex constitutes a part of the DMN, and plays a role in alternating activated and deactivated mental states to respond to demanding tasks^65^. Interestingly, the SMG, precuneus, and cerebellum have also been shown to exhibit a differential pattern that allows the distinction of violent individuals due to their importance in regulating and inhibiting inappropriate behaviors^16,18^. Thus, we would like to conclude that these patterns might underlie a diminished ability to cope with novelty and stressors, which partly explains dropout. That is, a reduced ability to integrate new experiences and, in turn, cope with them or respond appropriately might explain why these men feel overwhelmed by the content of interventions or accepting their new situation.

### Recidivism

Our results have revealed that only a few brain regions explain recidivism. Contrary to our expectations, the TOFusC and OFusG, located in the occipital lobe, emerged from all brain areas as the important ones for explaining recidivism. We hypothesized that the parietal and temporal lobes and the cerebellum would explain recidivism, as concluded by Delfin et al.^24^. Positive rsFc associations were found between those occipital brain regions and the aITG right, cerebellum Crus1 and vermis 9 (cerebellum). Therefore, our data partly sustained the conclusions of Delfin et al.^24^ given that the cerebellum was involved in explaining recidivism. Differences across studies could be attributed to the methodology applied in each study, with our study focusing on rsFC and Delfin only assessing the activation of isolated brain areas during a resting period. In any case, the regions that emerged in our study are involved in multimodal sensory integration^66^ and planning abilities or working memory^67^, such as that involved in facial processing^59^. These patterns could be applied to the altered cognitive processing of IPV perpetrators, which in turn, facilitate violent intake or being involved in antisocial behaviors^68^.

### Limitations

Despite the interest of the conclusions of this research, it is necessary to pay attention to several limitations that affect the external validity of these conclusions, and which could guide future research in this field. The main limitation is the relatively reduced sample size and the relative homogeneity of the sample (mainly based on Spanish men). Thus, it would be necessary to replicate the results with a larger sample size and combine samples across countries, as well as introduce 'sex' as an important variable for future research. In this sense, not only would it be necessary to include samples of men, but also others of violent women. Second, the inherent limitation of applying a ROI-based approach should be kept in mind. In other words, measuring brain activity with multiple divisions diminishes our ability to create a whole vision of the brain voxel-wise. Furthermore, it considerably increases the risk of type 1 error. However, we applied Bonferroni corrections to reduce the risk. Third, it would be particularly important to consider the temporal stability of these rsFC patterns, which would help demonstrate whether we can use them as a reliable "trait" to characterize violent individuals. Unfortunately, a relatively recent systematic review^13^ pointed out that a scarce number of studies, all of them cross-sectional, have measured rsFC in violent individuals.

## Conclusions

Our research reinforces the importance of combining biological markers, such as neuroimaging techniques, with psychological instruments to adequately explain, at least in part, the treatment compliance and recidivism of IPV perpetrators. In this sense, rsFC should be considered a complementary measurement to those conclusions provided by self-reports and qualitative interviews to explain this complex phenomenon. Moreover, this resting measurement obtained with fMRI should be incorporated into other experimental paradigms which submit participants to specific tasks. This would enable us to properly understand how participants change from a resting period to a demanding task and how they return to “normality” after ending it. Last, the model that explains treatment compliance and recidivism was able to better establish the subtypes of IPV perpetrators based on several variables collected with self-reports and qualitative interviews^68^. Therefore, future steps should consider whether rsFC, in combination with the variables mentioned above, facilitates the establishment of IPV subtypes for a good prediction of these key variables. In this regard, future empirical research should consider all these questions to reinforce neuroimaging results.

## Data Availability

The datasets generated and/or analysed during the current study are not publicly available but are available from the corresponding author on reasonable request.

## References

[1] Sardinha, L., Maheu-Giroux, M., Stöckl, H., Meyer, S. R. & García-Moreno, C. Global, regional, and national prevalence estimates of physical or sexual, or both, intimate partner violence against women in 2018. *Lancet* **399**(10327), 803–813. 10.1016/S0140-6736(21)02664-7 (2022). 35182472 10.1016/S0140-6736(21)02664-7 PMC8885817

[2] Expósito-Álvarez, C., Santirso, F. A., Gilchrist, G., Gracia, E. & Lila, M. Participants in court-mandated intervention programs for intimate partner violence perpetrators with substance use problems: A systematic review of specific risk factors. *Psychosoc. Interv.* **32**(2), 89–108. 10.5093/pi2023a7 (2023). 37383646 10.5093/pi2023a7 PMC10294470

[3] Neilson, E. C., Gulati, N. K., Stappenbeck, C. A., George, W. H. & Davis, K. C. Emotion regulation and intimate partner violence perpetration in undergraduate samples: A review of the literature. *Trauma Violence Abuse* **24**(2), 576–596. 10.1177/15248380211036063 (2023). 34551642 10.1177/15248380211036063 PMC8938307

[4] Aharoni, E. *et al.* Neuroprediction of future rearrest. *Proc. Natl. Acad. Sci.* **110**(15), 6223–6228. 10.1073/pnas.1219302110 (2013). 23536303 10.1073/pnas.1219302110 PMC3625297

[5] Glenn, A. L. & Raine, A. Neurocriminology: Implications for the punishment, prediction and prevention of criminal behaviour. *Nat. Rev. Neurosci.* **15**(1), 54–63. 10.1038/nrn3640 (2014). 24326688 10.1038/nrn3640

[6] Lamsma, J., Mackay, C. & Fazel, S. Structural brain correlates of interpersonal violence: Systematic review and voxel-based meta-analysis of neuroimaging studies. *Psychiatry Rese. Neuroimaging* **267**, 69–73. 10.1016/j.pscychresns.2017.07.006 (2017). 10.1016/j.pscychresns.2017.07.006 PMC5670119 28772208

[7] Raine, A. A neurodevelopmental perspective on male violence. *Infant Mental Health J.* **40**(1), 84–97. 10.1002/imhj.21761 (2019). 10.1002/imhj.21761 30586472

[8] Brenner, P. S. & DeLamater, J. Lies, damned lies, and survey self-reports? Identity as a cause of measurement bias. *Soc. Psychol. Q.* **79**(4), 333–354. 10.1177/0190272516628298 (2016). 29038609 10.1177/0190272516628298 PMC5639921

[9] Cole, M. W., Ito, T., Cocuzza, C. & Sanchez-Romero, R. The functional relevance of task-state functional connectivity. *J. Neurosci.* **41**(12), 2684–2702. 10.1523/JNEUROSCI.1713-20.2021 (2021). 33542083 10.1523/JNEUROSCI.1713-20.2021 PMC8018740

[10] Tran, S. M. *et al.* Task-residual functional connectivity of language and attention networks. *Brain Cogn.* **122**, 52–58. 10.1016/j.bandc.2018.02.003 (2018). 29471283 10.1016/j.bandc.2018.02.003 PMC6015731

[11] Grafton, S. T. & Volz, L. J. From ideas to action: The prefrontal–premotor connections that shape motor behavior. *Handb. Clin. Neurol.* **163**, 237–255. 10.1016/B978-0-12-804281-6.00013-6 (2019). 31590733 10.1016/B978-0-12-804281-6.00013-6

[12] Romero-Martínez, Á., Bressanutti, S. & Moya-Albiol, L. A systematic review of the effectiveness of non-invasive brain stimulation techniques to reduce violence proneness by interfering in anger and irritability. *J. Clin. Med.* **9**(3), 882. 10.3390/jcm9030882 (2020). 32213818 10.3390/jcm9030882 PMC7141522

[13] Romero-Martínez, Á. *et al.* The brain resting-state functional connectivity underlying violence proneness: Is it a reliable marker for neurocriminology? A systematic review. *Behav. Sci.* **9**(1), 11. 10.3390/bs9010011 (2019). 30650635 10.3390/bs9010011 PMC6359496

[14] Yang, Y. & Raine, A. Prefrontal structural and functional brain imaging findings in antisocial, violent, and psychopathic individuals: A meta-analysis. *Psychiatry Res. Neuroimaging* **174**(2), 81–88. 10.1016/j.pscychresns.2009.03.012 (2009). 10.1016/j.pscychresns.2009.03.012 PMC2784035 19833485

[15] Nikolic, M., Pezzoli, P., Jaworska, N. & Seto, M. C. Brain responses in aggression-prone individuals: A systematic review and meta-analysis of functional magnetic resonance imaging (fMRI) studies of anger- and aggression-eliciting tasks. *Prog. Neuro-Psychopharmacol. Biol. Psychiatry* **119**, 110596. 10.1016/j.pnpbp.2022.110596 (2022). 10.1016/j.pnpbp.2022.110596 35803398

[16] Leutgeb, V. *et al.* Altered cerebellar-amygdala connectivity in violent offenders: A resting-state fMRI study. *Neurosci. Lett.* **610**, 160–164. 10.1016/j.neulet.2015.10.063 (2016). 26523791 10.1016/j.neulet.2015.10.063

[17] Varkevisser, T., Gladwin, T. E., Heesink, L., van Honk, J. & Geuze, E. Resting-state functional connectivity in combat veterans suffering from impulsive aggression. *Soc. Cogn. Affect. Neurosci.* **12**, 1881–1889. 10.1093/scan/nsx113 (2017). 29040723 10.1093/scan/nsx113 PMC5716169

[18] Chen, C. *et al.* Regional homogeneity of resting-state brain abnormalities in violent juvenile offenders: A biomarker of brain immaturity?. *J. Neuropsychiatry Clin. Neurosci.* **27**, 27–32. 10.1176/appi.neuropsych.13030044 (2015). 25716485 10.1176/appi.neuropsych.13030044

[19] Sun, Q., Zhang, Y., Zhou, J. & Wang, X. Altered resting-state functional connectivity in the default mode network in male juvenile violent offenders. *Brain Imaging Behav.* **16**(2), 608–616. 10.1007/s11682-021-00535-3 (2022). 34480692 10.1007/s11682-021-00535-3 PMC9010331

[20] Amaoui, S., Marín-Morales, A., Martín-Pérez, C., Pérez-García, M. & Verdejo-Román, J. Social mentalizing in male perpetrators of intimate partner violence against women is associated with resting-state functional connectivity of the Crus II. *J. Psychiatr. Res.* **150**, 264–271. 10.1016/j.jpsychires.2022.03.044 (2022). 35427824 10.1016/j.jpsychires.2022.03.044

[21] Amaoui, S. *et al.* Resting-state functional connectivity and socioemotional processes in male perpetrators of intimate partner violence against women. *Sci. Rep.* **12**(1), 1–11. 10.1038/s41598-022-14181-2 (2022). 35710854 10.1038/s41598-022-14181-2 PMC9203491

[22] Arce, R., Arias, E., Novo, M. & Fariña, F. Are interventions with batterers effective? A meta-analytical review. *Psychosoc. Interv.* **29**(3), 153–164. 10.5093/pi2020a11 (2020).

[23] Karakurt, G., Koç, E., Çetinsaya, E. E., Ayluçtarhan, Z. & Bolen, S. Meta-analysis and systematic review for the treatment of perpetrators of intimate partner violence. *Neurosci. Biobehav. Rev.* **105**, 220–230. 10.1016/j.neubiorev.2019.08.006 (2019). 31415863 10.1016/j.neubiorev.2019.08.006 PMC6742529

[24] Delfin, C. *et al.* Prediction of recidivism in a long-term follow-up of forensic psychiatric patients: Incremental effects of neuroimaging data. *PLoS One* **14**(5), e0217127. 10.1371/journal.pone.0217127 (2019). 31095633 10.1371/journal.pone.0217127 PMC6522126

[25] Zhutovsky, P. *et al.* Individual prediction of trauma-focused psychotherapy response in youth with posttraumatic stress disorder using resting-state functional connectivity. *NeuroImage Clin.* **32**, 102898. 10.1016/j.nicl.2021.102898 (2021). 34911201 10.1016/j.nicl.2021.102898 PMC8645516

[26] Baeza-Delgado, C. *et al.* A practical solution to estimate the sample size required for clinical prediction models generated from observational research on data. *Eur. Radiol. Exp.* **6**(1), 22. 10.1186/s41747-022-00276-y (2022). 35641659 10.1186/s41747-022-00276-y PMC9156610

[27] Lila, M., Gracia, E. & Catalá-Miñana, A. Individualized motivational plans in batterer intervention programs: A randomized clinical trial. *J.Consult. Clin. Psychol.* **86**(4), 309. 10.1037/ccp0000291 (2018). 29648853 10.1037/ccp0000291

[28] Muñoz-Rivas, M. J., Andreu Rodríguez, J. M., Graña-Gómez, J. L., O’Leary, D. K. & González, M. P. Validation of the modified version of the conflict tactics scale (M-CTS) in a Spanish population of youths. *Psicothema* **19**, 693–698 (2007). 17959128

[29] Straus, M. A., Hamby, S. L., Boney-McCoy, S. & Sugarman, D. B. The revised conflict tactics scales (CTS2): Development and preliminary psychometric data. *J. Family Issues* **17**, 283–316. 10.1177/019251396017003001 (1996).

[30] Puig, J. *et al.* Resting-state functional connectivity magnetic resonance imaging and outcome after acute stroke. *Stroke* **49**(10), 2353–2360. 10.1161/STROKEAHA.118.021319 (2018). 30355087 10.1161/STROKEAHA.118.021319 PMC6645916

[31] Kaufman, A. & Kaufman, N. *K-BIT Test Breve de Inteligencia [K-BIT Brief Intelligence Test]* (TEA, 1997).

[32] Bowers, T. L. & Pantle, M. L. Shipley institute for living scale and the Kaufman Brief Intelligence Test as screening instruments for intelligence. *Assessment* **5**(2), 187–195. 10.1177/107319119800500209 (1998). 9626394 10.1177/107319119800500209

[33] Romero-Martínez, Á., Lila, M., Sarrate-Costa, C., Comes-Fayos, J. & Moya-Albiol, L. neuropsychological performance, substance misuse, and recidivism in intimate partner violence perpetrators. *Psychosoc. Interv.* **32**(2), 69–77. 10.5093/pi2022a7 (2023). 37383645 10.5093/pi2022a7 PMC10294454

[34] Romero-Martínez, Á., Lila, M., Sarrate-Costa, C., Comes-Fayos, J. & Moya-Albiol, L. The interaction between attention deficit hyperactivity disorder and neuropsychological deficits for explaining dropout and recidivism of intimate partner violence perpetrators. *Eur. J. Psychol. Appl. Leg. Context* **15**(1), 33–42. 10.5093/ejpalc2023a4 (2023).

[35] Saunders, J. B., Aasland, O. G., Babor, T. F., De la Fuente, J. R. & Grant, M. Development of the alcohol use disorders identification test (AUDIT): WHO collaborative project on early detection of persons with harmful alcohol consumption-II. *Addiction* **88**(6), 791–804. 10.1111/j.1360-0443.1993.tb02093.x (1993). 8329970 10.1111/j.1360-0443.1993.tb02093.x

[36] Contell-Guillamón, C., Gual-Solé, A. & Colom-Farran, J. Test para la identificación de trastornos por uso de alcohol (AUDIT): Traducción y validación del AUDIT al catalán y castellano (in Spanish) [Test for the identification of disorders due to alcohol use (AUDIT): Translation and validation of the AUDIT into Catalan and Spanish]. *Adicciones* **11**, 337–347. 10.20882/adicciones.613 (1999).

[37] Allen, J. P., Litten, R. Z., Fertig, J. B. & Babor, T. A review of research on the Alcohol Use Disorders Identification Test (AUDIT). *Alcoholism Clin. Exp. Res.* **21**(4), 613–619. 10.1111/j.1530-0277.1997.tb03811.x (1997). 9194913

[38] Lila, M., Gracia, E. & Catalá-Miñana, A. More likely to dropout, but what if they don’t? Partner violence offenders with alcohol abuse problems completing batterer intervention programs. *J. Interpersonal Violence* **35**(9–10), 1958–1981. 10.1177/0886260517699952 (2020). 10.1177/0886260517699952 29294698

[39] Vitoria-Estruch, S., Romero-Martínez, A., Lila, M. & Moya-Albiol, L. Differential cognitive profiles of intimate partner violence perpetrators based on alcohol consumption. *Alcohol* **70**, 61–71. 10.1016/j.alcohol.2018.01.006 (2018). 29800781 10.1016/j.alcohol.2018.01.006

[40] Miele, G. M., Carpenter, K. M., Cockerman, M. S., Trautman, K. D. & Baline, J. Substance use severity scale (SDSS): Reliability and validity of a clinician-administered interview for DSM-IV substance use disorders. *Drug Alcohol Use* **59**, 63–75. 10.1016/s0376-8716(99)00111-8 (2000). 10.1016/s0376-8716(99)00111-8 10706976

[41] Vélez-Moreno, A. *et al.* Adaptación al español de la substance use severity scale: Resultados preliminares [Spanish adaptation of the substance dependence severity scale: Preliminar results]. *Adicciones* **25**(4), 339–347 (2013). 24217503

[42] Kaye, S. & Darke, S. Determining a diagnostic cut-off on the Severity of Dependence Scale (SDS) for cocaine dependence. *Addiction* **97**(6), 727–731. 10.1046/j.1360-0443.2002.00121.x (2002). 12084142 10.1046/j.1360-0443.2002.00121.x

[43] van der Pol, P. *et al.* Reliability and validity of the Severity of Dependence Scale for detecting cannabis dependence in frequent cannabis users. *Int. J. Methods Psychiatr. Res.* **22**(2), 138–143. 10.1002/mpr.1385 (2013). 23670783 10.1002/mpr.1385 PMC6878253

[44] Andrés-Pueyo, A., López, S. & Álvarez, E. Valoración del riesgo de violencia contra la pareja por media de la SARA [*Assessment of the risk of intimate partner violence and the SARA*]. *Papeles del Psicólogo* **29**(1), 107–122 (2008).

[45] Kropp, P. R., Hart, S. D., Webster, C. W. & Eaves, D. *Manual for the Spousal Assault Risk Assessment Guide* 2nd edn. (British Columbia Institute on Family Violence, 1995).

[46] Messing, J. T. & Thaller, J. The average predictive validity of intimate partner violence risk assessment instruments. *J. Interpersonal Violence* **28**(7), 1537–1558. 10.1177/0886260512468250 (2013). 10.1177/0886260512468250 23262817

[47] Benjamini, Y. & Hochberg, Y. Controlling the false discovery rate: A practical and powerful approach to multiple testing. *J. R. Stat. Soc. Ser. B (Methodol.)* **57**(1), 289–300. 10.1111/j.2517-6161.1995.tb02031.x (1995).

[48] Woo, C. W., Krishnan, A. & Wager, T. D. Cluster-extent based thresholding in fMRI analyses: Pitfalls and recommendations. *Neuroimage* **91**, 412–419. 10.1016/j.neuroimage.2013.12.058 (2014). 24412399 10.1016/j.neuroimage.2013.12.058 PMC4214144

[49] Pedregosa, F. *et al.* Scikit-learn: Machine learning in Python. *J. Mach. Learn. Res.* **12**, 2825–2830 (2011).

[50] Kuppachi, G. H. R. Probabilistic and machine learning enhancement to CONN toolbox. *Master’s Projects*10.31979/etd.r748-y3qw (2020).

[51] Potvin, S., Giguère, C. É. & Mendrek, A. Functional connectivity during visuospatial processing in schizophrenia: A classification study using lasso regression. *Neuropsychiatr. Dis. Treat.* **17**, 1077–1087. 10.2147/NDT.S304434 (2021). 33888984 10.2147/NDT.S304434 PMC8055358

[52] Dowden, C. & Brown, S. L. The role of substance abuse factors in predicting recidivism: A meta-analysis. *Psychol. Crime Law* **8**(3), 243–264. 10.1080/10683160208401818 (2002).

[53] Koehler, J. A., Humphreys, D. K., Akoensi, T. D., Sánchez de Ribera, O. & Lösel, F. A systematic review and meta-analysis on the effects of European drug treatment programmes on reoffending. *Psychol. Crime Law* **20**(6), 584–602. 10.1080/1068316X.2013.804921 (2014).

[54] Whitfield-Gabrieli, S. & Nieto-Castanon, A. Conn: A functional connectivity toolbox for correlated and anticorrelated brain networks. *Brain Connect.* **2**(3), 125–141. 10.1089/brain.2012.0073 (2012). 22642651 10.1089/brain.2012.0073

[55] De Benedictis, A. *et al.* Infra-occipital supra-tentorial approach for resection of low-grade tumor of the left lingual gyrus: 2-dimensional operative video. *Oper. Neurosurg.* **21**(3), E257–E258. 10.1093/ons/opab172 (2021). 10.1093/ons/opab172 34022047

[56] Suzuki, W. L. & Amaral, D. G. Perirhinal and parahippocampal cortices of the macaque monkey: Cortical afferents. *J. Comp. Neurol.* **350**(4), 497–533. 10.1002/cne.903500402 (1994). 7890828 10.1002/cne.903500402

[57] Lee, T. M., Chan, S. C. & Raine, A. Hyperresponsivity to threat stimuli in domestic violence offenders: A functional magnetic resonance imaging study. *J. Clin. Psychiatry* **70**(1), 36. 10.4088/jcp.08m04143 (2009). 19192464 10.4088/jcp.08m04143

[58] Romanski, L. M. & LeDoux, J. E. Equipotentiality of thalamo-amygdala and thalamo-cortico-amygdala circuits in auditory fear conditioning. *J. Neurosci.* **12**(11), 4501–4509. 10.1523/JNEUROSCI.12-11-04501.1992 (1992). 1331362 10.1523/JNEUROSCI.12-11-04501.1992 PMC6575992

[59] Pessoa, L., McKenna, M., Gutierrez, E. & Ungerleider, L. G. Neural processing of emotional faces requires attention. *Proc. Natl. Acad. Sci.* **99**(17), 11458–11463. 10.1073/pnas.172403899 (2002). 12177449 10.1073/pnas.172403899 PMC123278

[60] O’Neil, E. B., Hutchison, R. M., McLean, D. A. & Köhler, S. Resting-state fMRI reveals functional connectivity between face-selective perirhinal cortex and the fusiform face area related to face inversion. *Neuroimage* **92**, 349–355. 10.1016/j.neuroimage.2014.02.005 (2014). 24531049 10.1016/j.neuroimage.2014.02.005

[61] Rigon, A., Voss, M. W., Turkstra, L. S., Mutlu, B. & Duff, M. C. Relationship between individual differences in functional connectivity and facial-emotion recognition abilities in adults with traumatic brain injury. *NeuroImage Clin.* **13**, 370–377. 10.1016/j.nicl.2016.12.010 (2017). 28123948 10.1016/j.nicl.2016.12.010 PMC5222957

[62] Senkans, S., McEwan, T. E. & Ogloff, J. R. Conceptualising intimate partner violence perpetrators’ cognition as aggressive relational schemas. *Aggress. Violent Behav.* **55**, 101456. 10.1016/j.avb.2020.101456 (2020).

[63] Burgess, P. W., Dumontheil, I. & Gilbert, S. J. The gateway hypothesis of rostral prefrontal cortex (area 10) function. *Trends Cogn. Sci.* **11**(7), 290–298. 10.1016/j.tics.2007.05.004 (2007). 17548231 10.1016/j.tics.2007.05.004

[64] Dumontheil, I., Burgess, P. W. & Blakemore, S. J. Development of rostral prefrontal cortex and cognitive and behavioural disorders. *Dev. Med. Child Neurol.* **50**(3), 168–181. 10.1111/j.1469-8749.2008.02026.x (2008). 18190537 10.1111/j.1469-8749.2008.02026.x PMC2488407

[65] Busler, J. N., Yanes, J. A., Bird, R. T., Reid, M. A. & Robinson, J. L. Differential functional patterns of the human posterior cingulate cortex during activation and deactivation: A meta-analytic connectivity model. *Exp. Brain Res.* **237**(9), 2367–2385. 10.1007/s00221-019-05595-y (2019). 31292696 10.1007/s00221-019-05595-y PMC7577043

[66] Onitsuka, T. *et al.* Middle and inferior temporal gyrus gray matter volume abnormalities in chronic schizophrenia: An MRI study. *Am. J. Psychiatry* **161**(9), 1603–1611. 10.1176/appi.ajp.161.9.1603 (2004). 15337650 10.1176/appi.ajp.161.9.1603 PMC2793337

[67] Schmahmann, J. D. The cerebellum and cognition. *Neurosci. Lett.* **688**, 62–75. 10.1016/j.neulet.2018.07.005 (2019). 29997061 10.1016/j.neulet.2018.07.005

[68] Romero-Martínez, Á., Lila, M., Gracia, E., Martín-Fernández, M. & Moya-Albiol, L. Generally antisocial batterers with high neuropsychological deficits present lower treatment compliance and higher recidivism. *Psychol. Violence* **11**(3), 318. 10.1037/vio0000296 (2021).

